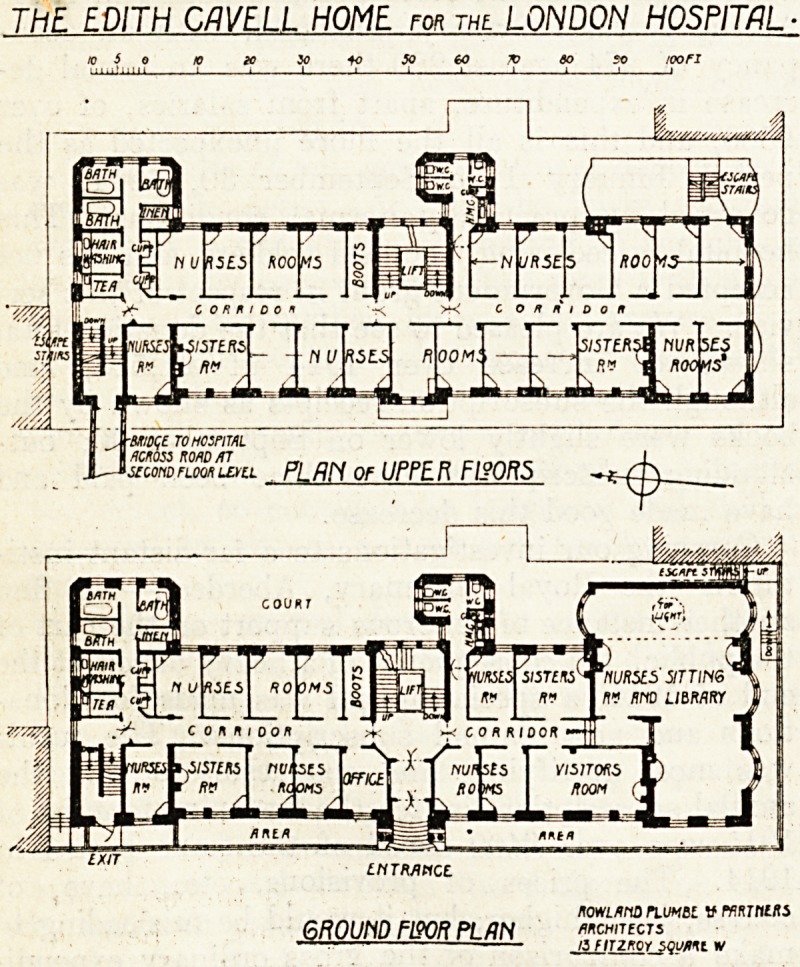# Edith Cavell Home for Nurses: London Hospital

**Published:** 1916-01-15

**Authors:** 


### HOSPITAL ARCHITECTURE AND CONSTRUCTION.

### Edith Cavell Home for Nurses: London Hospital.

This addition to the already extensive accommodation for nurses at the London Hospital was, when first projected, to be called the "Alexandra Home," after the president of the hospital, H.M. Queen Alexandra. It is now, by desire of the Royal president, to be named after the noble woman who sacrificed her life in Belgium in the cause of humanity.

THE EDITH CAVELL HOME FOR THE LONDON HOSPITAL·

The building, which is eight storeys high including the basement, occupies a site at the corner of Eastmount Street and Oxford Street. The site is very restricted in area, and very little free space is left around the building.

The basement contains the heating apparatus, servants' quarters, and stores. On the ground floor are the main entrance from the street, offices, visitors' room, a large sitting-room for nurses, two sisters' rooms, 10 nurses' rooms, and bathrooms, lavatories, etc. The upper floors are all alike, and contain on each floor two sisters' rooms and twenty nurses' rooms; the total accommodation being for twelve sisters and 110 nurses. On each floor, besides an ample supply of sanitary offices and bathrooms, there is a hair-washing room fitted with an electric blower for drying the hair, also a boot-cleaning room and a small room for making tea. The sisters' rooms, which apparently are meant to be bed-sitting rooms, are each provided with a fireplace, and all the rooms have fitted wardrobes. Besides the central staircase, in the well of which is an electric lift, there are two escape staircases, one at each end of the building.

The attic floor is fitted up as a boxroom, and contains also iron lockers for the use of each nurse. On the second floor the home has direct communication with the hospital by means of a bridge over Eastmount Street. By means of this the nurses have access to the dining-room in the old building opposite.

The architects for the building were Messrs. Rowland Plumbe and Partners.

## Figures and Tables

**Figure f1:**